# Intracellular symbiosis of algae with possible involvement of mitochondrial dynamics

**DOI:** 10.1038/s41598-017-01331-0

**Published:** 2017-04-27

**Authors:** Chihong Song, Kazuyoshi Murata, Toshinobu Suzaki

**Affiliations:** 1 Graduate School of Science, Kobe University, 1-1 Rokkodai, Nada, Kobe 657-8501 Japan; 2 National Institute for Physiological Sciences, 5-1 Higashiyama Myodaiji, Okazaki, Aichi 444-8787 Japan; 3 National Institute for Physiological Sciences, 5-1 Higashiyama Myodaiji, Okazaki, Aichi 444-8787 Japan

## Abstract

Algal endosymbiosis is widely present among eukaryotes including many protists and metazoans. However, the mechanisms involved in their interactions between host and symbiont remain unclear. Here, we used electron microscopy and three-dimensional reconstruction analyses to examine the ultrastructural interactions between the symbiotic zoochlorella and the organelles in the host *Paramecium bursaria*, which is a model system of endosymbiosis. Although in chemically fixed samples the symbiotic algae show no direct structural interactions with the host organelles and the perialgal vacuole membrane (PVM), in cryofixed *P*. *bursaria* samples the intimate connections were identified between the host mitochondria and the symbiotic algae via the PVM. The PVM was closely apposed to the cell wall of the symbiotic algae and in some places it showed direct contacts to the host mitochondrial membrane and the cell wall of the symbiotic algae. Further, the PVM-associated mitochondria formed a mitochondrial network and were also connected to host ER. Our observations propose a new endosymbiotic systems between the host eukaryotes and the symbionts where the benefiting symbiosis is performed through intimate interactions and an active structural modification in the host organelles.

## Introduction

A number of algae live in cells of protists and invertebrates such as *Porifera* and *Cnidaria* ^1, 2^. A recent study also reported that algae invade and live in symbiosis with embryonic salamander tissues and cells^3^. Algal symbionts obtain nitrogen and carbon dioxide from the host cells and provide the host with photosynthetic products^1–6^. These symbioses are thought to be beneficial for both the symbiont and the host. For example, intracellular zoochlorella (unicellular green algae, Chloroplastida) in Paramecia is protected from *Chlorella* viruses by living in the host^7, 8^ and it contributes to the host’s tolerance to environmental changes, such as temperature fluctuations and ultraviolet rays^9, 10^. Furthermore, symbiosis with algae is necessary for healthy growth of salamander embryos^3^. For these reasons, intracellular symbiosis of algae is a good example for explaining the ecological success of mixotrophic associations and the environmental adaptation^2, 11^.

The green ciliate *Paramecium bursaria* (superphylum: Alveolata) is a microorganism that can accommodate several hundred zoochlorella cells in its cytoplasm. The host and the symbiotic zoochlorella can be cultured discretely and the endosymbiotic relationship can be re-established simply by co-culturing the two organisms^12–14^. Aposymbiotic *P*. *bursaria* can also be infected artificially with other microorganisms, including *Scenedesmus* (green algae), several species of yeast, and bacteria^15, 16^. In light of these facts, mixotrophic ciliates such as *P*. *bursaria* are possible intermediates between producers and consumers. Therefore, they are regarded as important ecological links in aquatic ecosystems and also as crucial contributors to the formation of biodiversity in the aquatic environment^2^. *P*. *bursaria* is one of the well-studied objects among such mixotrophic ciliates, and is an excellent model for studying endosymbiosis^4–17^.

Early electron microscopy (EM) studies of *P*. *bursaria* showed that symbiotic algal cells are usually enclosed within a perialgal vacuole membrane (PVM)^18^. Similar studies also demonstrated that intracellular algal cells in metazoa are normally surrounded by a symbiosomal membrane, which is thought to be equivalent to the PVM^1–3^. These membranes prevent digestion of the symbionts in the host’s cytoplasm and control the mutual exchange of various substances between the two partners^19–22^.

Symbiotic algae, which are protected from lysosomal attack with their surrounding membrane, are analogous in this respect to the apicomplexan parasite *Toxoplasma gondii*. *T*. *gondii* is a protozoan pathogen that infects mammalian cells and can proliferate inside the host cells within a specialized non-fusogenic membrane called the parasitophorous vacuole membrane (here we call PaVM)^23^. Like the PVM of symbiotic algae, *T*. *gondii* exchanges nutrients and metabolites with its host through the PaVM^24^. However, unlike symbiotic algae, the pores in the PaVM surrounding *T*. *gondii* allow rapid exchange of small molecules between the host and the parasites^25^. Another unusual characteristic of symbiotic *T*. *gondii* is that its surrounding membrane forms tight associations with the host’s mitochondria and endoplasmic reticulum (ER)^24, 26^; these associations seem to be important for nutrient acquisition^27^. However, an EM study demonstrated that symbiotic zoochlorellae in Paramecia did not form any associations with host organelles^19^. A number of EM studies of endosymbiotic systems have been performed to date, but none has reported direct interactions between symbiotic algae and host organelles^3, 28–32^.

Here, three-dimensional (3-D) ultrastructural EM analyses of cryofixed samples were employed to examine the structural relationships between the symbiotic zoochlorellae and the host organelles of *P*. *bursaria*. These experiments revealed for the first time the presence of direct contacts between ER-networked mitochondria in *P*. *bursaria* and symbiotic zoochlorella cells through their surrounding PVMs.

## Results

### Comparison of the chemical fixation and cryofixation methods

**Figure 1:**
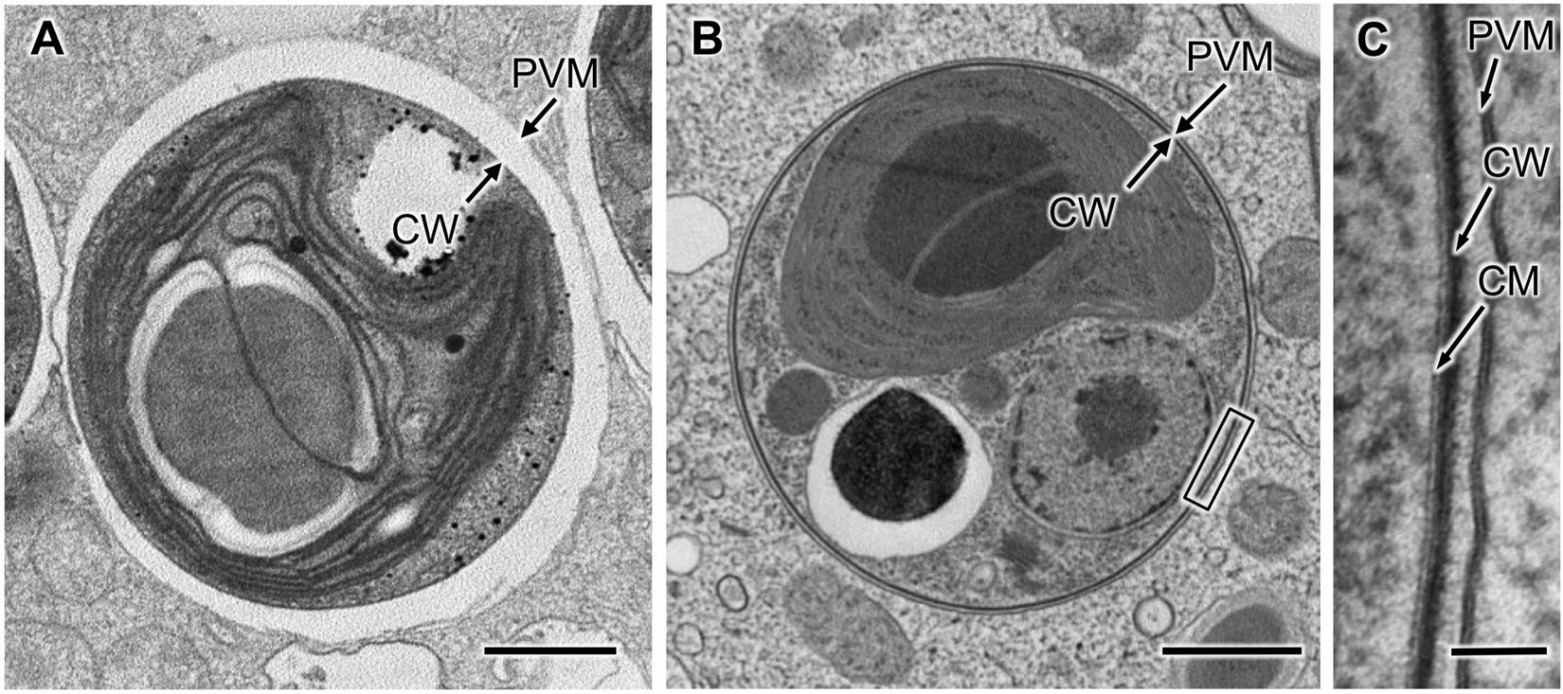
Ultrastructure of the PVM in chemically fixed and cryofixed samples. (**A**,**B**) TEM images of chemically fixed (**A**) and cryofixed (**B**) *P*. *bursaria* cells. (**C**) Enlargement of the boxed area shown in (**B**). Scale bars: 1 μm (**A**,**B**) and 50 nm (**C**). CM, cell membrane; CW, cell wall; PVM, perialgal vacuole membrane.

Transmission electron microscopy (TEM) was used to examine *P*. *bursaria* cells prepared using two different fixation methods: chemical fixation (3% glutaraldehyde and 1% OsO_4_) and cryofixation (cold metal-block freezing) followed by freeze substitution (1% OsO_4_). Cells prepared using these methods had different spatial relationships between the PVMs and zoochlorellae in the host cytoplasm. In chemically fixed cells, the PVM was physically separated from the cell wall of the symbiotic zoochlorella (Fig. 1A), while the PVM in cryofixed cells was positioned very close to the cell wall of the symbiotic zoochlorella (Fig. 1B,C). The distances between the cell wall and the PVM in chemically fixed cells (n = 300) and cryofixed cells (n = 300) were approximately 180 nm and 25 nm, respectively. Samples with sparse cytoplasm, such as plant cells with large vacuoles, often shrink during the dehydration process that follows chemical fixation^33^, suggesting that the extended distance between the symbiotic zoochlorellae and the PVM in chemically fixed specimens was caused through the preparation process^34^. From these results, all the following observations were carried out using cryofixation. However, we could not see any cross-linking structures between the symbiotic algae and the host cytoplasm in both samples. The results described above suggest that, despite the presence of the PVM, which completely separates symbiotic algae from the host’s cytoplasm, the distance from the symbiont is small enough to allow direct molecular interactions of the host’s organelles with symbiotic algae.

### Structures of subcortical region in *P*. *bursaria*

**Figure 2:**
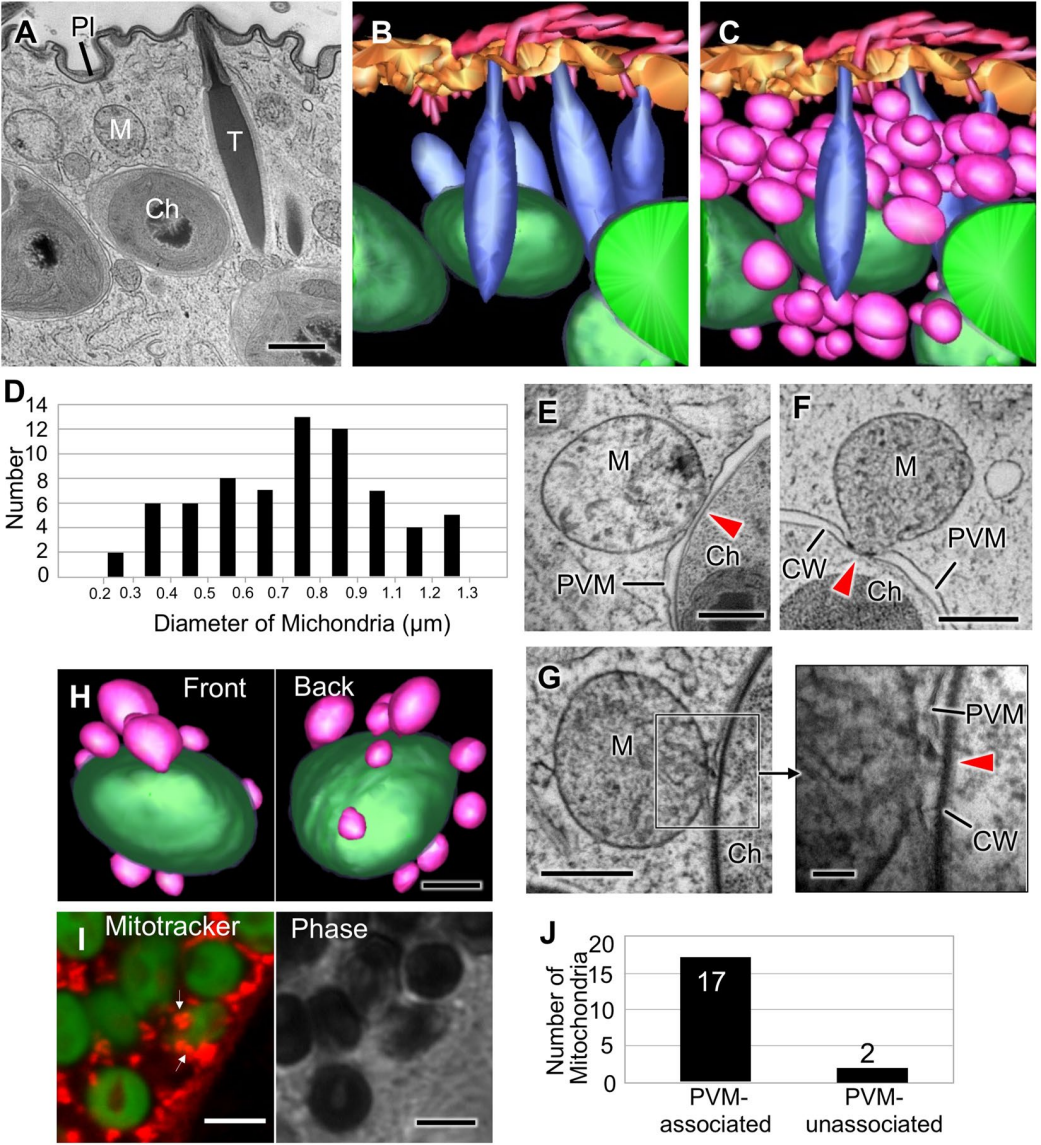
TEM analyses of the associations between *P*. *bursaria* mitochondria and symbiotic zoochlorella. (**A**) TEM image of the 31st of 45 serial ultrathin sections of a *P*. *bursaria* cell. (**B**,**C**) A 3-D reconstruction and segmentation of the subcortical region of *P*. *bursaria* generated from TEM images of 45 serial ultrathin sections with and without mitochondria, respectively. Symbiotic zoochlorella are shown in green, trichocysts are shown in blue, and mitochondria are shown in pink. (**D**) The distribution of mitochondrial diameters in the 3-D reconstruction. (**E**) TEM image of a mitochondrion attached to the PVM surrounding a zoochlorella cell. The arrowhead indicates the contacted area. (**F**) TEM image of a mitochondrion connected to the cell wall of a symbiotic zoochlorella. The arrowhead indicates the connecting area. (**G**) TEM image of the interconnecting bridge (arrowhead) between a mitochondrion and the cell wall of a zoochlorella cell. (**H**) A 3-D model of zoochlorella-associated mitochondria. (**I**) Fluorescence microscopy image of autofluorescencing zoochlorella cells (green) and MitoTraker Red-stained mitochondria (red) in *P*. *bursaria*. The arrows indicate the host mitochondria (shown in red) associated with symbiotic zoochlorella (shown in green). A corresponding phase contrast image is shown in the right panel. (**J**) The number of PVM-associated and -unassociated mitochondria within distance of 20 nm from the PVM in 3D model of Fig. 2C. Scale bars: 1 μm (**A**,**H**), 500 nm (**E**–**G**), 100 nm (inset in **G**), and 5 μm (**I**). Ch, zoochlorella; CW, cell wall; Pl, plasma membrane; M, mitochondrion; PVM, perialgal vacuole membrane; T, trichocyst.

Electron tomography using high-voltage EM allows the observation of 2–4 μm thick sections. Since zoochlorellae are 3–7 µm in diameter, high-voltage electron tomography does not allow direct observation of a whole zoochlorella cell. Therefore, images of serial thin sections of *P*. *bursaria* were used to generate a 3-D reconstruction. Cryofixed *P*. *bursaria* cells were embedded in Spurr’s resin and cut into 100 nm thick sections. A whole symbiotic zoochlorella cell and its surrounding organelles could be visualized within 45 serial sections. Figure 2A shows the image of the 31st section. The total sampling volume of the *P*. *bursaria* cell reconstructed from the serial sections was approximately 162 μm^3^ (6.0 μm × 6.0 μm × 4.5 μm). Within the volume, the cell cortex was approximately 135 μm^3^ (Fig. 2B,C and Supplementary Movie 1). In the whole serial sections, 11th to 40th contained a single zoochlorella cell (the central green structures shown in Fig. 2B and Supplementary Movie 1). The largest diameter of the zoochlorella observed from a number of consecutive slices was approximately 3 μm. In spite that zoochlorella cells are nearly spherical^34^, the 3D-reconstruction image of the zoochlorella cells appeared ellipsoidal in shape. This discrepancy is likely owing to compression of the sections during cutting with a diamond knife^35^. *P*. *bursaria* possesses a number of extrusive organelles named trichocysts, which are thought to be involved in cell defense against predator^36^. In the 3-D reconstruction, the trichocysts (blue structures in Fig. 2B) were approximately 1 μm wide and 4–5 μm long, and were docked beneath the *P*. *bursaria* plasma membrane. Each zoochlorella cell was observed to be surrounded by multiple numbers of trichocysts, thus partially restricting its free horizontal transfer along the inner surface of the plasma membrane (Supplementary Movie 1). Previous reports on the ultrastructure of other non-symbiotic species of *Paramecium* showed that subcortical areas are mainly filled with trichocysts and mitochondria without zoochlorella^37, 38^.

### Associations between mitochondria and zoochlorellae

A number of mitochondria were located close to the zoochlorella in *P*. *bursaria* (pink structures in Fig. 2C and Supplementary Movie 1). In the 3-D reconstruction, 70 mitochondria of various sizes (mean diameter, 0.83 ± 0.23 μm) were identified (Fig. 2D), and the total volume of the mitochondria was 17.7 μm^3^, corresponding to 13% of the subcortical space we observed. Distinct structural connections between the mitochondria and the PVM or the zoochlorella were identified in 17 mitochondria. 11 of them were attached to the PVM (Fig. 2E and Supplementary Fig. 1A), and the other 6 were closely associated with the cell wall of the symbiotic zoochlorella via the PVM (Fig. 2F and Supplementary Fig. 1B). Shallow depressions on the surface of the zoochlorella cell wall were also identified (arrowhead in Fig. 2F) to which outer membranes of the mitochondria were tightly associated, suggesting that the zoochlorellae were strongly associated with the surrounding mitochondria. In other cases, the outer membranes of the mitochondria were tightly associated with the PVM and looked connected to the cell walls of zoochlorellae (Fig. 2G). Most of the mitochondria located in the vicinity of the symbiotic zoochlorella were associated with the zoochlorella cells or the PVM (Fig. 2H and Supplementary Movie 2), and only a small number of them, despite in great proximity, were not directly associated with the PVM (Fig. 2J).

Fluorescence microscopy analysis of MitoTracker Red-stained *P*. *bursaria* also confirmed the localization of multiple mitochondria around the symbiotic zoochlorella cells that were detected by autofluorescent signals shown in green (Fig. 2I).

**Figure 3:**
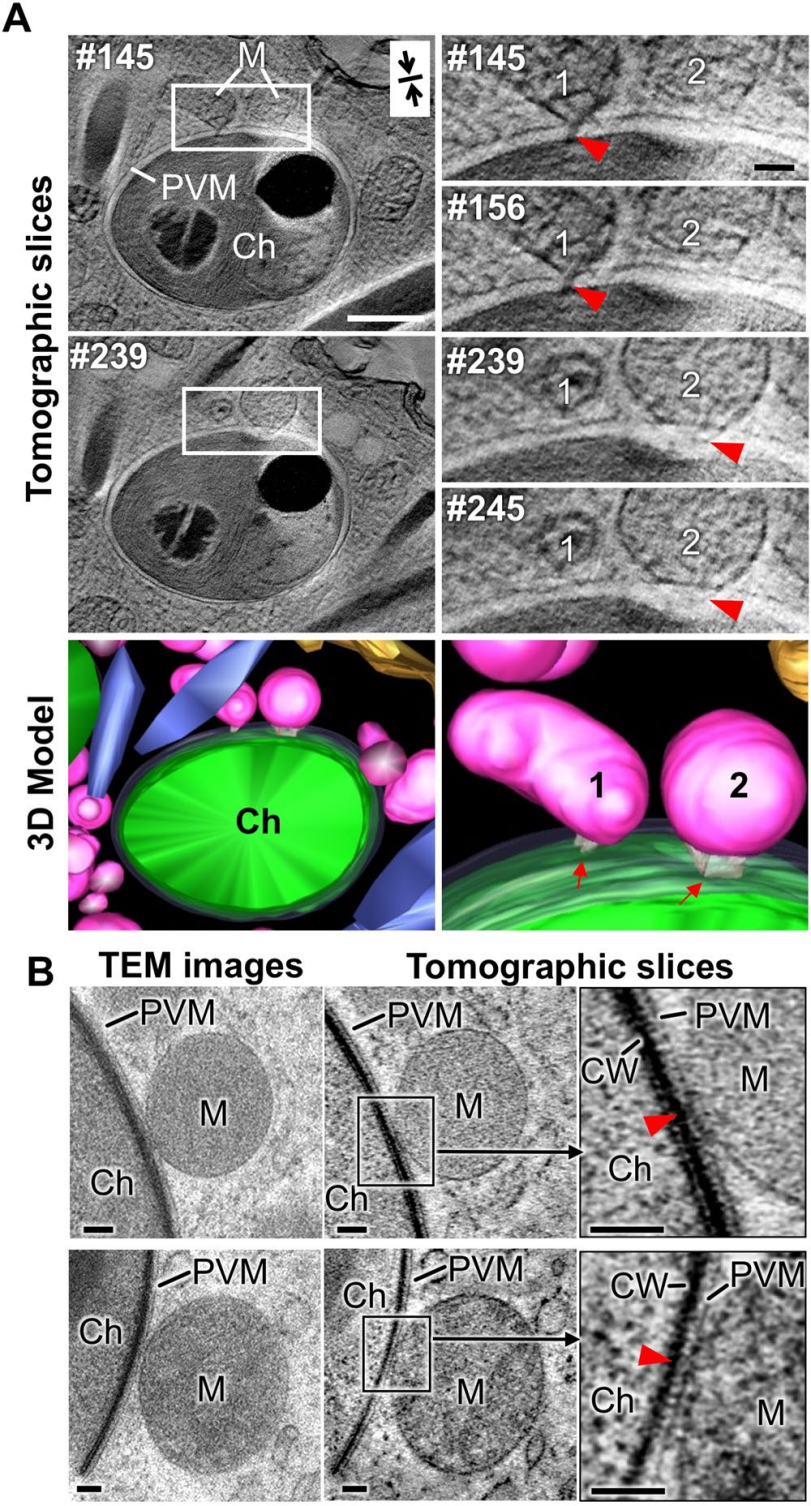
Electron tomography analyses of the associations between *P*. *bursaria* mitochondria and symbiotic zoochlorella. (**A**) Representative XY slices through a high-voltage electron tomogram of a *P*. *bursaria* cell (divided into 400 tomographic slices) and a 3-D model reconstructed from the images (left panels). The thickness of each level is approximately 2.5 nm. The tomograms on the upper right panel show close-ups of the boxed areas in the tomograms on the left panels. Two black arrows indicate the orientation of the compression when cut with a diamond knife. Mitochondrion number 1 and the cell wall of the zoochlorella are joined by an interconnecting bridge (red arrowhead in 145th and 156th levels). Some substances seem to be present between mitochondrion number 2, which is attached to the PVM, and the cell wall of zoochlorella (red arrowhead in 239th and 245th levels). A hollow area that seems to be influenced by the adjacent mitochondrion is present in the cell wall of the zoochlorella. In the 3-D model, zoochlorellae are shown in green and mitochondria are shown in pink. The translucent region represents PVM. (**B**) TEM images of a 200-nm-thick section (left) and their tomographic slices (middle and right). The thickness of each level in the slices is approximately 0.9 nm. The interconnecting substance is shown between the mitochondria and the cell wall of the zoochlorella (red arrowheads). Scale bars: 500 nm (white), 100 nm (black). Ch, zoochlorellae; CW, cell wall; M, mitochondria; PVM, perialgal vacuole membrane.

To generate a more accurate and higher resolution image of the mitochondrial contact site with symbiotic zoochlorella, electron tomography was employed. A 1-μm-thick section of *P*. *bursaria* was resolved into 400 tomographic slices (Supplementary Movie 3) by electron tomography using a high-voltage EM and each slice was examined with sufficient image contrast and with high resolution (2.5 nm/pixel in z-axis). The results suggested the existence of linking or sticking structures between PVM-associated mitochondria and the cell wall of zoochlorellae (Fig. 3A and Supplementary Movie 4). Hollow regions in the zoochlorella cell wall were also identified at the sites mitochondria attached (mitochondria 2 in Fig. 3). In order to analyze the detailed structure, we also operated the electron tomography with a 200-nm-thick section. The tomographic slices at higher resolution (~0.9 nm/pixel in z-axis) than the 1-μm-thick section showed clearly the existence of the structural connection between the host mitochondria and the cell wall of zoochlorella (Fig. 3B and Supplementary Movies 5 and 6).

A 4-μm-thick section was also examined by the same procedure of the 1-μm-thick section. The sample was slightly damaged by the growth of ice crystals, but the tomographic slices show that many host mitochondria are in contact with the symbiotic zoochlorella or the PVM. We found and reconstructed 36 mitochondria located within 500 nm (averaged radius of mitochondria is ~400 nm) from the surface of symbiotic zoochlorella. The 3D model shows that 70% and more of mitochondria are in direct contact with the PVM. This result indicates that the host mitochondria actively interact with symbiotic zoochlorella itself or the PVM surrounding it. The similar statistical result from serial sections is also shown in Fig. 2J.

### Network formation among mitochondria

**Figure 4:**
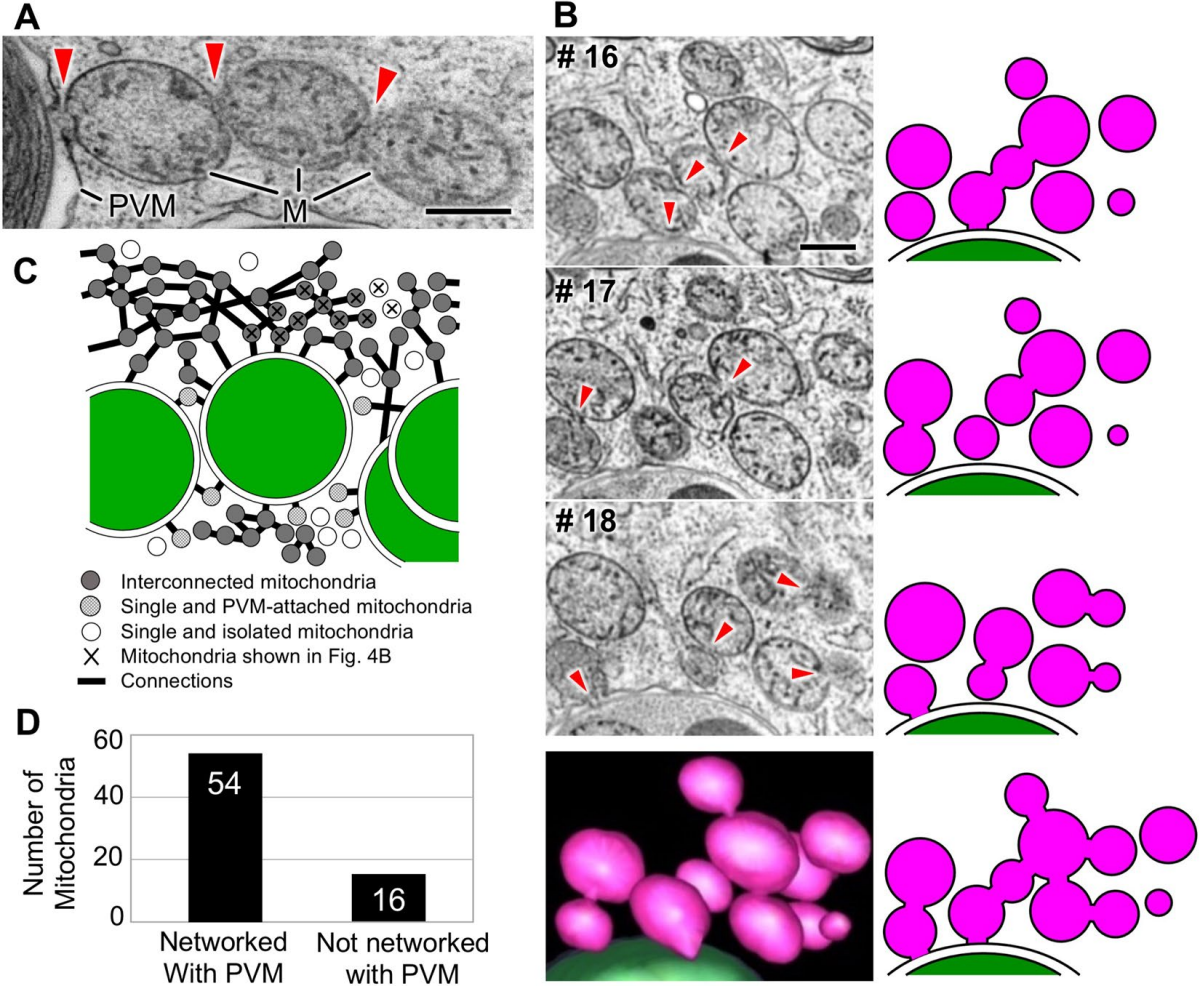
The mitochondrial network around the symbiotic zoochlorella in *P*. *bursaria*. (**A**) A TEM image of the mitochondrial network associated with the PVM in *P*. *bursaria*. The arrowheads indicate the connections between adjacent mitochondria. (**B**) TEM images of the mitochondrial network in the 15th, 16th, and 17th ultrathin sections of *P*. *bursaria*, and the corresponding 2-D models of this region of the cell. The arrowheads indicate the connections between adjacent mitochondria. The lower panel shows segmentation in 3-D reconstruction and its corresponding 2-D image. The mitochondria and the symbiotic zoochlorellae are colored by pink and green, respectively. (**C**) A schematic drawing of the direct connections formed by all 70 mitochondria present in the 3-D model. (**D**) A histogram showing the numbers of mitochondria that are directly or indirectly associated with the PVM (shown as filled and shaded circles in Fig. 4C), and those that are not incorporated in the PVM-associated mitochondrial network (shown as open circles in Fig. 4C). Scale bars: 500 nm. M, mitochondrion; PVM, perialgal vacuole membrane.

The TEM analyses of *P*. *bursaria* demonstrated that mitochondria are highly concentrated in the subcortical region of the cell (Fig. 2C and Supplementary Fig. 2). The images also indicated that mitochondria are firmly associated each other via membrane fusion or strong contacts to form a beaded or moniliform appearance (Fig. 4A). Based on these observations, we hypothesized that mitochondria establish an elaborate network in the subcortical region. To determine the number of mitochondria that are connected to each other, the spatial relationship of all mitochondria in the observed area was examined using a 3-D reconstructed model. Figure 4B shows a series of micrographs from the 16th, 17th, and 18th serial sections, in which 11 mitochondria were present. When examining the single sections, only two or three mitochondria appeared to be connected. However, the 3-D analysis indicated that most of the mitochondria were actually connected with their neighbors (Fig. 4B and Supplementary Movie 6). The 70 mitochondria present in the 3-D model were illustrated in a 2-D map (Fig. 4C). More than 75% of the mitochondria were connected to each other (Fig. 4D), and the connected mitochondria formed a network and surrounded the zoochlorellae.

### Associations between mitochondria and ER

**Figure 5:**
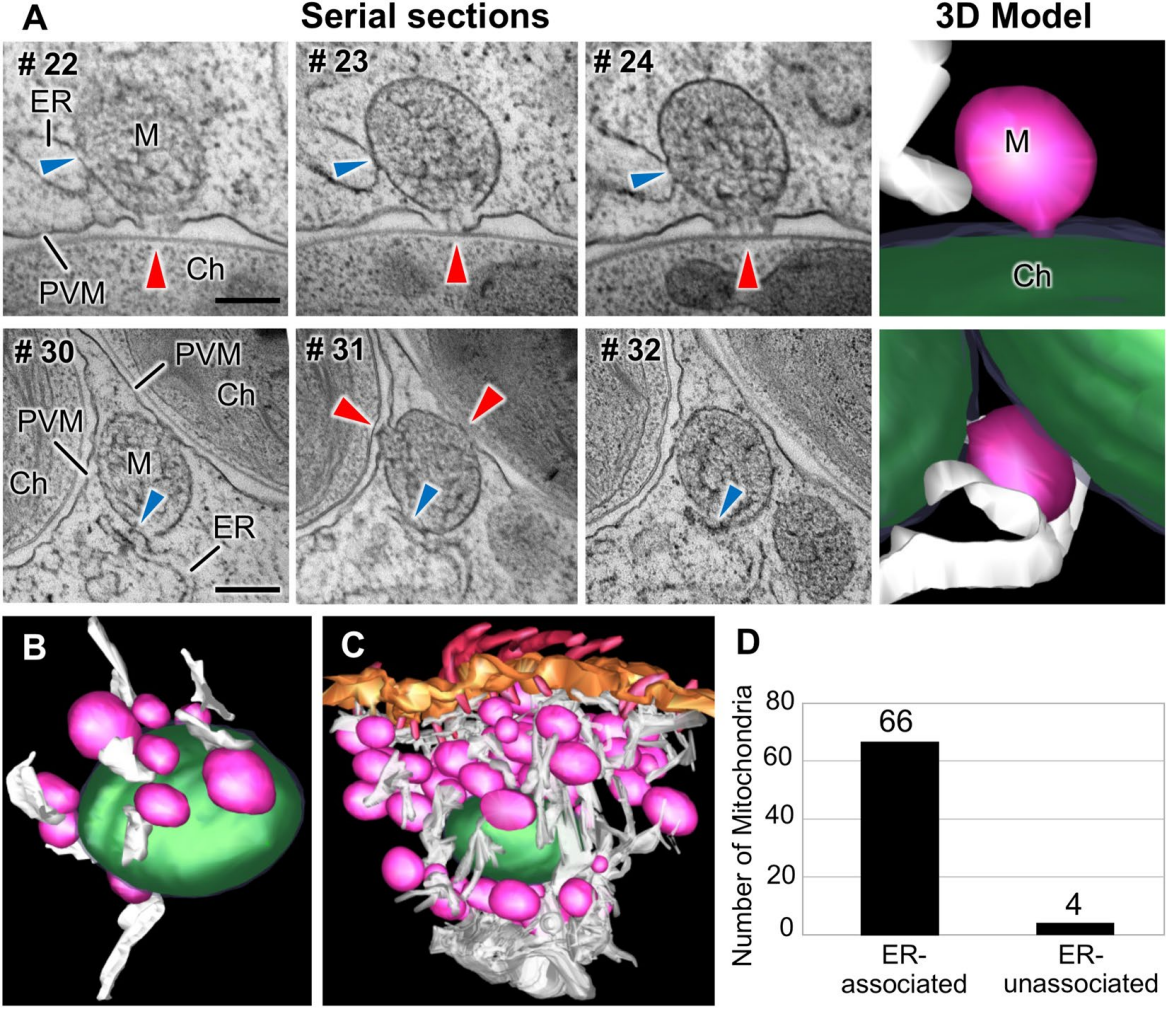
Connections between mitochondria and the ER in *P*. *bursaria*. (**A**) TEM images of serial sections of *P*. *bursaria* showing the associations between mitochondria, ER, and symbiotic zoochlorella through the PVM. The red and blue arrowheads indicate the sites at which mitochondria are connected to the symbiotic zoochlorella and ER, respectively. The panels on the right show the 3-D segmentation models of the mitochondrial connections; in these images, mitochondria, zoochlorellae, and ER are colored by pink, green, and white, respectively. Scale bars: 500 nm. (**B**) A 3-D segmentation model showing that all of the mitochondria associated with the symbiotic zoochlorella are connected to the ER. (**C**) A full volume 3-D model showing the network of mitochondria connected with ER at the subcortical area of *P*. *bursaria*. (**D**) The numbers of ER-associated and -unassociated mitochondria in the full volume 3-D model. Ch, zoochlorella; ER, endoplasmic reticulum; M, mitochondrion; PVM, perialgal vacuole membrane.

The relationship between the host ER and the symbiotic zoochlorella was investigated. In the 3-D model, all PVM-associated mitochondria were connected to ER, while direct interaction between the host ER and the PVM was rarely observed (Fig. 5B and Supplementary Movie 1). A quantitative analysis showed that 66 of the 70 mitochondria present in the model were in contact with ER (Fig. 5C,D). Membrane continuities between ER and mitochondria have been reported in many cell types (from fungi to mammals) and these associations have clearly defined functions in the transport of lipids and Ca^2+^ between ER and mitochondria^39^. All PVM-associated mitochondria were connected to ER, in addition to the high frequency of connections between ER and mitochondria. These results suggest that the presence of mitochondria, working in collaboration with ER, would be necessary to promote the establishment of zoochlorella symbiosis in the sub-cortical region of the host ciliate.

## Discussion

TEM observations of cryofixed samples revealed that the PVM in *P*. *bursaria* was apposed closely to the cell wall of the symbiotic zoochlorella with a constant gap of approximately 20 nm. By contrast, preparation of *P*. *bursaria* using the conventional chemical fixation method caused an obvious shrinkage of the sample, resulting in detachment of the PVM from the cell wall of the symbiotic zoochlorella. These results suggest that chemical fixation of samples is not an appropriate method for examination of the spatial relationship between a host’s cytoplasm and its symbiont. This relationship is important in understanding the role of the perialgal space in the exchange of materials between the host and the symbiont. Experiments have shown that zoochlorella release large amounts of maltose beyond the cell membrane and the cell wall under acidic conditions^20, 21, 40^. The low pH of the perialgal space is considered to further facilitate the transport of maltose from the perialgal space to the host’s cytoplasm by a H^+^/maltose co-transporter^41, 42^. However, Rands *et al*. reported that the density of colloidal-gold-labeled DAMP (a pH indicator) in the perialgal space of green cnidarian hydra was as low as that in the cytoplasmic matrix, and thus suggested that the interior of the perialgal vacuole in green cnidarian hydra was almost neutral^43^. They performed the immunoelectron microscopy analyses with chemically fixed samples; there is thus the possibility that the symbiotic zoochlorella were shrunken by the chemical fixation procedure. Based on our observations in the cryo-fixed samples, the actual space between the zoochlorella and the PVM was approximately 7-fold smaller than that reported by Rands *et al*. According to this estimation, the 7-hold lower DAMP density value Rands *et al*. reported may be an artifact caused by perialgal space expansion during EM processing.

Close apposition of the PVM to the cell wall of algae allows the host’s organelles to interact directly with the symbiont. In this study, we identified several structural contacts between mitochondria and zoochlorella or its enclosing PVM. We often observed that mitochondria in other protozoan or invertebrate hosts, such as cnidarian hydras, heliozoans, and amoebas, are directly associated with symbiotic algae. Many morphological studies have attempted to identify structural aspects of algae symbionts in various hosts^3, 28–32^; however, to our knowledge, there has been only two reports describing a relationship between host mitochondria and symbiotic algae. Kerney *et al*. reported that intracellular algae are often located in close proximity to mitochondria in salamander embryos^3^. In addition, Feng *et al*. showed an electron micrograph showing a mitochondrion in close proximity to symbiotic algal PVM^32^. Although the features and functions of these mammalian intracellular algae and *symbiodinium* differ from those described in protozoan or invertebrate hosts, these observations indicate that mitochondrial association is a common feature of endosymbiotic algae.

With our microscopic observation alone it is difficult to understand the function of the interaction between host mitochondria and symbiotic algae. However, we can discuss about this by comparing with the interactions between the host organelles and the PaVM of Apicomplexan parasites^26^. Like the PVM of symbiotic zoochlorella, the PaVM plays an important role in protecting Apicomplexan parasites from lysosomal attack and exchanging nutrients and metabolites with the host^24^. In addition, similar to our observation on the PVM, PaVM forms tight associations with host mitochondria and ER^24, 26^. However, the Apicomplexan parasitic endosymbiotic systems show some morphological differences from those of the symbiotic zoochlorella: (1) Intracellular Apicomplexa are situated relatively far from the PaVM; (2) The PaVM shows a direct association with ER while, in the case of symbiotic zoochlorella, the PVM-ER association is mediated by mitochondria; and (3) Apicomplexa are not directly associated with the host mitochondria unlike the symbiotic zoochlorella.

Some studies on Apicomplexa have suggested that the host mitochondria provide essential nutrients to the parasite via interactions between the mitochondria and the PaVM. Crawford *et al*. revealed the lack of the mitochondrial lipoate synthase gene in the parasitic *T*. *gondii*, suggesting that the host is providing the lipase to the parasite through a direct interaction of the host mitochondria with the PaVM^44^. The PaVM of *Encephalitozoon cuniculi*, an obligate intracellular pathogen belonging to the phylum Microsporidia, is also associated closely with its host’s mitochondria^45^. Because *E*. *cuniculi* does not possess the tricarboxylic acid cycle and the respiratory chain^46^, it has been suggested that the ATPs in this pathogen are supplied by the host mitochondria. In contrast, in the *Paramecium*-*Chlorella* system, the symbiotic zoochlorellae still possesse an ability to live independently without the host. This implies that their interactions with the host’s mitochondria are not basically required for obtaining trophic factors that are indispensable for their growth. However, zoochlorella cells in endosymbiotic condition grow faster than those in free-living condition^47^. There may be a possibility that the host mitochondria contribute to the growth of zoochlorella, in part, by supplying the materials to the inside of the PVM.

The PVM of zoochlorellae in *P*. *bursaria* is formed from the food vacuole membrane^14, 48^. After the formation of the PVM, it needs to grow and divide in accordance with the growth and division of the symbiotic zoochlorellae. The mechanisms of the formation of the PVM from the food vacuole membrane in *P*. *bursaria*, including the growth and division, have not been characterized yet. In contrast, it has been studied more in Apicomplexa. After invasion of its mammalian host by *T*. *gondii*, the proteins in the phagosomal membrane that initially surrounds the parasite are actively exchanged to the parasites’ novel proteins, which are delivered from the parasite’s rhoptries (secretory organelles), and the dense granules from the parasites are also inserted into the membrane^49^. After this, the lipids are provided from the host to enable growth of the newly formed PaVM^50, 51^. Sinai *et al*. suggested a potential model for the trafficking of lipids from both mitochondria and ER to PaVM via their direct contacts^27^.

Associations of membranes between ER and mitochondria have been reported in various cell types; it is well known that lipids are transferred from ER to mitochondria via this association, where phosphatidylserine is converted to phosphatidylethanolamine by the mitochondrial enzyme, phosphotidylserine decarboxylase^52, 53^. Hailey *et al*. suggested that mitochondria supply autophagosomal membranes, where the mitochondrial lipids were replenished from ER via direct connections between these organelles^54^. These findings demonstrate that direct membrane contact between different organelles facilitates the transfer of lipids. The EM images presented here showed that the PVM surrounding an algal cell in *P*. *bursaria* formed an elaborate network of mitochondria with ER. It is also verified from the fact that the contents of the lipids in the PVM are similar with those of the mitochondria.

In addition to their roles of supplying materials to and from symbionts, we found the host mitochondria have a unique function for the stabilization of symbiotic zoochlorella. Localization of zoochlorella cells in the subcortical area of the host is essential for maintaining symbiosis stably^18, 19, 55^. In contrast, zoochlorella cells located deep in the cytoplasm or involved in cytoplasmic streaming are destined to be digested. Studies of the ciliate *Tetrahymena thermophila* showed that the majority of mitochondria were located close to the plasma membrane and were aligned along the ciliary rows^56^. EM study by Włoga *et al*. showed that mitochondria in *T*. *thermophila* are associated with neighboring mitochondria and plasma membranes where septins are localized, suggesting that septins are involved in the regulation of mitochondrial dynamics and the association of mitochondria with other organelles in the ciliate *Tetrahymena* ^57^. Septin has been identified also in *P*. *bursaria* ^57, 58^. We have observed that mitochondria of *P*. *bursaria* are associated not only with symbiotic zoochlorellae but also with trichocysts and the plasma membrane (Supplementary Fig. 3). The large mitochondrial network may function as a structural scaffold that stabilizes the symbiotic zoochlorellae at the subcortical region where digestive activity in the cytoplasm is suppressed, thereby allowing the zoochlorellae to protect from lysosomal attack.

In summary, this study describes the morphological features of symbiotic algae in *P*. *bursaria*. The data presented here suggested that the host mitochondria and ER are involved in the organization of intracellular algal symbiosis in the cytoplasm. Our observations propose a new endosymbiotic system between the eukaryote hosts and the symbionts where the benefitting symbiosis is performed through intimate interactions and an active structural modification in the host organelles.

## Methods

### Materials

The German strain of *P*. *bursaria* (strain PB-SW1) was provided by Prof. Hans-Dieter Görtz (Stuttgart University, Germany). The cells were cultured in bacteria-free monoxenic medium and *Chlorogonium capillatum* was used as a food source, as described previously^13^.

### Cryofixation and freeze substitution

The cells were collected by low-speed centrifugation and suspensions with high cell densities were cryofixed by slamming them onto a liquid nitrogen-cooled (−196 °C) copper block by using a metal contact quick freezing device (VFZ-101, Japan Vacuum Device, Japan). The frozen materials were transferred to cold (−80 °C) acetone containing 1% osmium tetroxide and substitution was performed by incubation at −80 °C for 72 h. The temperature was then manually elevated in a stepwise manner (−20 °C for 1 h, 4–10 °C for 0.5 h, and then room temperature for 0.5 h). The materials were washed with 100% acetone at room temperature and embedded in Spurr’s resin (Polysciences, Inc.). Polymerization of the resin was performed at 70 °C for 8 h. The ultrathin sections were stained with 3% uranyl acetate and lead citrate.

### Chemical fixation

The cells were prefixed at room temperature for 10 min with a low osmotic pressure fixation fluid (3% glutaraldehyde in 50 mM Na-cacodylate buffer (pH 7.0) containing 20 μM MgSO_4_ and 2 μM sucrose) to avoid shrinkage and swelling of the cells or the organelles^34^. The fixed materials were washed three times with the same buffer and then postfixed at room temperature for 30 min with 1% OsO_4_ in the same buffer. The fixed cells were dehydrated through a graded ethanol series (50%, 70%, 90%, 95%, 99%, and 100%) and embedded in Spurr’s resin at 70 °C for 8 h. Ultrathin sections were stained with 3% uranyl acetate and lead citrate.

### 3-D reconstruction from serial sections

After the material was fixed using the freeze-substitution fixation method, serial ultrathin sections (approximately 100 nm thick) were prepared using a diamond knife (EM UC7 ultramicrotome; Leica, Austria). Forty-five serial ultrathin sections were successfully collected onto Formvar-coated grids (Cu, one slot). The sections were contrasted by staining with 2% uranyl acetate and lead citrate, and were examined using an H-7100 transmission electron microscope (Hitachi, Japan) operating at 75 kV. The images taken at a magnification of 10 k were recorded in 1 k × 1 k CCD camera (C4741-95; Hamamatsu Photonics) at a pixel size of 9.43 nm. The 3-D reconstructions were generated using IMOD software version 4.7.15^59^. Organelle volumes were determined using the IMODINFO program. The image segmentation in the 3-D reconstructions was performed with IMOD software.

### Electron tomography

The specimens prepared by freeze-substitution fixation method were also subjected to high-voltage EM observations. Thick sections (1and 4 μm) were obtained using a glass knife and placed onto a single-slot grid. The grids were labeled with colloidal gold particles as a fiducial marker. A high-voltage electron microscope (H1250M; Hitachi) operating at 1,250 kV was used for data acquisition. Tilt series were recorded on SO-163 Kodak electron image films at 2° increments in a tilt range from −60° to +60°, respectively. The micrograms were developed for 12 min with a high strength of Kodak D-19 developer and digitized at a scanning step of 25 nm with Nikon Coolscan 9000ED. After image alignment using colloidal gold particles, the 3-D reconstruction was performed by weighted back-projection using IMOD software. The segmentation in the 3-D reconstructions was performed with IMOD and Amira version 5. 4. 5 (FEI Visualization Science Group, Burlington, MA, USA). In addition, a section of 200 nm thickness was imaged using a JEM2200FS electron microscope (JEOL Inc.) equipped with a field-emission electron source operated at 200 kV and an in-column (omega-type) energy filter operated in zero-energy-loss mode with a slit width of approximately 25 eV. Tilt series were recorded using a 4 k × 4 k CCD camera (F415, TVIPS, Germany) at 2° increments in a tilt range from −70° to +70°. The electron micrographs were taken at a magnification of 10,000 (9.36 Å/pixel). The 3-D reconstruction was performed by simultaneous iterative reconstruction technique using IMOD software.

### Fluorescence microscopy

The cells were incubated for 45 min in 0.01% Knop medium [0.24 mM Ca(NO_3_)_2_, 0.14 mM KNO_3_, 0.06 mM MgSO_4_, and 0.10 mM KH_2_PO_4_] containing 50 nM MitoTracker RedCMXRos (Invitrogen Molecular Probes). After three washes with 0.01% Knop medium, the labeled cells were fixed in 4% paraformaldehyde for 20 min, washed with phosphate-buffered saline, and analyzed with a confocal laser scanning microscope (FluoView FV-1000, excitation: 559 nm, emission range: 575–675 nm; Olympus Co. LTD).

## Electronic supplementary material

Supplementary information

Supplementary Movie 1

Supplementary Movie 2

Supplementary Movie 3

Supplementary Movie 4

Supplementary Movie5

Supplementary Movie 6

Supplementary Movie 7

Supplementary Movie 8
