# Supplementary material for: Intracellular symbiosis of algae with possible involvement of mitochondrial dynamics
Source: Sci Rep. 2017 Apr 27;7:1221. doi: 10.1038/s41598-017-01331-0 (PMC5430747; doi:10.1038/s41598-017-01331-0)
Supplement: Supplementary file 1 — Supplementary information [file 41598_2017_1331_MOESM1_ESM.pdf]

Supplementary information

Intracellular symbiosis of algae with possible involvement of mitochondrial dynamics

Chihong Song[1,*,†], Kazuyoshi Murata[2] and Toshinobu Suzaki[1,*]

[1] Graduate School of Science, Kobe University, 1-1 Rokkodai, Nada, Kobe 657-8501, Japan

[2] National Institute for Physiological Sciences, 5-1 Higashiyama Myodaiji, Okazaki, Aichi 444-8787, Japan

[*]For correspondence. E-mail: chsong@nips.ac.jp (CS); suzaki@kobe-u.ac.jp (TS)

[†]Present address: National Institute for Physiological Sciences, 5-1 Higashiyama Myodaiji, Okazaki, Aichi 444-8787, Japan

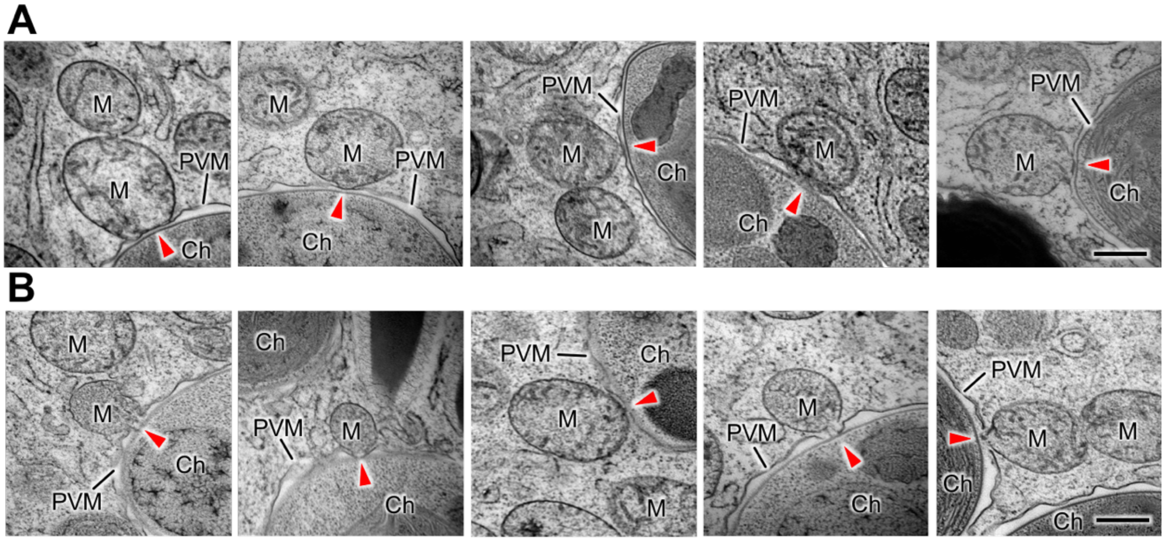

Supplementary Figure 1. Representative EM images of mitochondria associated with the symbiotic zoochlorella or the PVM in serial sections. (A) The host mitochondria associated with the PVM. (B) The host mitochondria associated with the cell wall of the symbiotic zoochlorella. The most approached images of mitochondria to the symbiotic zoochlorella or the PVM were individually selected in the serial sections. Red arrowheads indicate the connected areas. Scale bars: 500 nm. Ch, zoochlorella; M, mitochondrion; PVM, perialgal vacuole membrane.

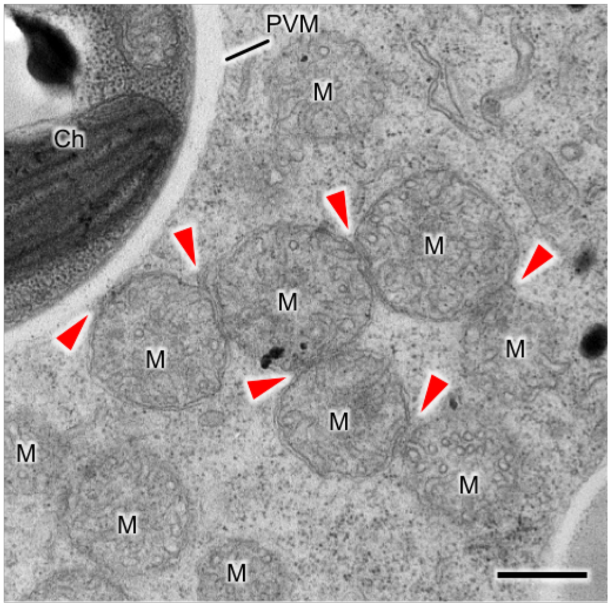

Supplementary Figure 2. Mitochondrial network observed in the chemical fixed specimen. Red arrowheads indicate the connected areas among mitochondria. Scale bar: 500 nm. Ch, zoochlorella; M, mitochondrion; PVM, perialgal vacuole membrane.

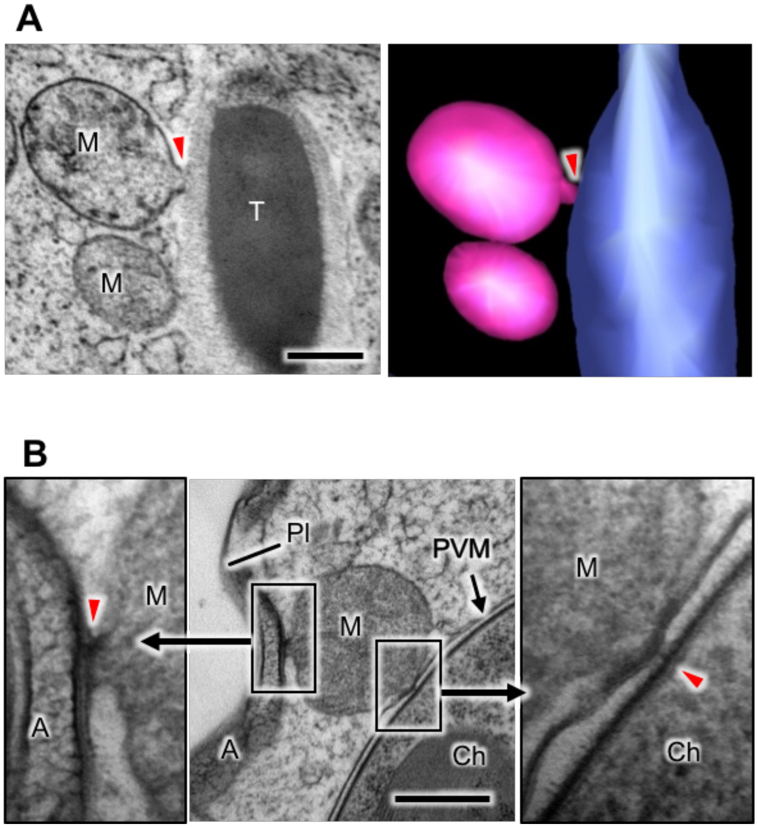

Supplementary Figure 3. Connections between mitochondria and other organelles in *P. bursaria*. (A) A mitochondrion (shown in pink in 3D model) is associated with a trichocyst (shown in blue in 3D model). (B) A mitochondrion (M) is associated with both the proximal surface of the alveoli and a PVM of the symbiotic zoochlorella. Red arrowheads indicate the connected areas. Scale bars: 500 nm. A, alveoli; Ch, zoochlorella; M, mitochondrion; Pl, Plasma membrane; PVM, perialgal vacuole membrane; T, trichocyst.

Supplementary Movie 1. 3D model of the subcortical area with a whole symbiotic zoochlorella in *P. bursaria.* Cilia, red; Host cell membrane, yellow; Mitochondria, pink; Perialgal vacuole membrane, translucence; Trichocysts, Blue; Zoochlorella, green.

Supplementary Movie 2. Segmentation of a symbiotic zoochlorella cell with its associated with the host mitochondria. Mitochondria, pink; Perialgal vacuole membrane, translucence; Zoochlorella, green.

Supplementary Movie 3. Tomographic slices of a 1 μm thick section generated by using high voltage electron tomography.

Supplementary Movie 4. Connecting site between the symbiotic zoochlorella and the host mitochondria visualized with high voltage EM. Mitochondria, pink; Perialgal vacuole membrane, translucence; Zoochlorella, green.

Supplementary Movie 5. Whole tomographic slices of the symbiotic zoochlorella and a host mitochondrion in a 200 nm-thick section shown in the top of Fig. 3B

Supplementary Movie 6. Whole tomographic slices of the symbiotic zoochlorella and a host mitochondrion in a 200-nm-thick section shown in the bottom of Fig. 3B

Supplementary Movie 7. Subcortical area of *P. bursaria* in a 4 μm thick section visualized by using high voltage electron tomography. We segmented 36 mitochondria located within

500 nm (averaged radius of mitochondria is ~ 400 nm) from the surface of symbiotic zoochlorella. Some mitochondria associate with the PVM and others don't associate with the PVM. Perialgal vacuole membrane (PVM), translucent skyblue; PVM-associated mitochondria, pink; PVM-unassociated mitochondria, brown.

Supplementary Movie 8. Mitochondrial network. Mitochondria, pink; Perialgal vacuole membrane, translucence; Zoochlorella, green.
